# Key Factors Associated With Nonuse of Telemedicine in Patients With Hypertension and/or Diabetes: Findings From the 2023 Indonesia Health Survey

**DOI:** 10.1155/ijta/5333547

**Published:** 2026-03-08

**Authors:** Sofa D. Alfian, Meliana Griselda, Mochammad A. A. Pratama, Imam A. Wicaksono, Raden M. Febriyanti, Widya N. Insani, Rizky Abdulah, Mahmathi Karuppannan

**Affiliations:** ^1^ Department of Pharmacology and Clinical Pharmacy, Faculty of Pharmacy, Universitas Padjadjaran, Jatinangor, Indonesia, unpad.ac.id; ^2^ Center of Excellence for Pharmaceutical Care Innovation, Universitas Padjadjaran, Jatinangor, Indonesia, unpad.ac.id; ^3^ Center for Health Technology Assessment, Universitas Padjadjaran, Jatinangor, Indonesia, unpad.ac.id; ^4^ Department of Biology Pharmacy, Faculty of Pharmacy, Universitas Padjadjaran, Jatinangor, Indonesia, unpad.ac.id; ^5^ Department of Pharmacy Practice and Clinical Pharmacy, Faculty of Pharmacy, Universiti Teknologi MARA Selangor Branch, Puncak Alam Campus, Bandar Puncak Alam, Malaysia; ^6^ Telehealth and Remote Patient Care Research Initiative Group, Universiti Teknologi MARA, Bandar Puncak Alam, Malaysia, uitm.edu.my

**Keywords:** diabetes, hypertension, telemedicine, the Indonesian Health Survey, usability

## Abstract

**Background:**

The Indonesian government has established a blueprint for health system digitalization aimed at improving health coverage. Despite the benefits of telemedicine services, its utilization remains low, and the factors associated with nonuse of telemedicine in Indonesia are not well understood.

**Objective:**

This study aimed to assess the prevalence of telemedicine use and to identify factors contributing to its nonuse among patients with hypertension and/or diabetes, particularly considering that these patients require long‐term medication management and monitoring.

**Methods:**

This national cross‐sectional study utilized data from the Indonesia Health Survey conducted in 2023, reflecting the postpandemic demographical conditions across 38 provinces in Indonesia. Telemedicine utilization and sociodemographic information were assessed based on a self‐reported questionnaire. Logistic regression was performed to identify sociodemographic factors associated with nonuse of telemedicine. Odds ratios (ORs) with 95% confidence intervals (CIs) were reported.

**Results:**

This study involved 63,012 patients with diabetes and/or hypertension. Most of them were women (65.1%), married (78.3%), aged 55–64 years (30.9%). Nearly all the respondents (98.0%) had not used telemedicine. Factors associated with nonuse of telemedicine included being unmarried (OR = 1.40; 95*%*CI = 1.11–1.77), older than 34 years (OR = 3.83; 95*%*CI = 1.90–7.73), having an educational background below the university level, farmer/fisherman and helper/laborer/driver, and living outside the islands of Java and Bali. Respondents with hypertension alone (OR = 1.67, 95*%*CI = 1.32–2.11) were more likely to report nonuse of telemedicine compared with those with both diabetes and hypertension.

**Conclusions:**

The usage of telemedicine among patients with hypertension and/or diabetes in Indonesia is low. Personalized approaches that consider patient‐specific factors and integrate telemedicine more frequently into the healthcare system are essential to enhance telemedicine adoption among patients with hypertension and/or diabetes in Indonesia.

## 1. Introduction

The burden of chronic diseases in Indonesia is a significant public health concern, as these conditions have become increasingly prevalent and pose serious challenges to the healthcare system [1]. Older populations typically experience chronic diseases [2]. However, they are now becoming more prevalent in the middle‐aged population [3] and even in young adults and adolescents [2, 4]. Chronic diseases, including diabetes mellitus, hypertension, cardiovascular disease, dyslipidemia, renal disease, and lung disease, account for a substantial number of deaths and disabilities across the country [2, 5]. In particular, diabetes mellitus and hypertension have emerged as the most prevalent chronic diseases, with incidence rates ranging from 0.9% to 3.4% for diabetes and 4.4% to 13.2% for hypertension across all provinces in Indonesia [2]. Therefore, addressing the burden of chronic diseases in Indonesia requires a multifaceted approach, including public health initiatives focused on education and awareness and improved access to healthcare services [6–8].

Digital health technology has shown promising improvements in health‐related outcomes in low‐to‐middle‐income countries [9]. Since the COVID‐19 outbreak catalyzed the adoption of telemedicine and digital healthcare in Indonesia, the Indonesian government has developed a blueprint for health system digitalization. A primary focus of this initiative is the expansion of telemedicine technology to enhance public familiarity with these services [10]. Digital health technologies in Indonesia are currently accessible through various telemedicine platforms provided by both public and private sectors, offering consultations, prescriptions, and referrals, often supported by partnerships with local healthcare providers [11]. Furthermore, the government envisions enhanced capabilities for disease mapping to improve prediction and prevention efforts, thereby advancing universal health coverage through telemedicine [10].

Patients with hypertension and diabetes are required to take medication regularly and undergo continuous monitoring over an extended period. Therefore, prioritizing the adoption of telemedicine for these patients may be crucial. Previous studies have reported positive impacts on disease management among patients with chronic diseases who utilized telemedicine [12–14], particularly among patients with hypertension and diabetes. Among 264 diabetic patients, 60.2% had poor glycemic control during the COVID‐19 pandemic, and those who used telemedicine were nearly twice as unlikely to have uncontrolled glycemia [15]. Furthermore, telemedicine has shown efficacy in providing online counseling and monitoring of blood pressure for hypertensive patients, leading to improved disease management [16].

Despite the benefits of telemedicine, studies have reported a low utilization rate among Indonesians [15, 17, 18], despite observations of a high willingness to use such services [19, 20]. Factors associated with the nonuse of telemedicine in some countries including Indonesia have been limited to small sample sizes [8, 21], often focusing solely on older age groups [22], providing minimal information on financial aspects [23], and primarily targeting the general public [8, 22, 24]. Exploring the potential factors that contribute to the nonuse of telemedicine among patients with hypertension and/or diabetes mellitus could be beneficial for improving the management of these chronic conditions in Indonesia. Therefore, this study is aimed at assessing the prevalence of telemedicine use and identifying factors contributing to its nonuse among patients with hypertension and/or diabetes in Indonesia.

## 2. Methods

This study is reported in accordance with the cross‐sectional study standard: Strengthening the Reporting of Observational Studies in Epidemiology (STROBE) (see Table S1. STROBE assessment).

### 2.1. Study Design and Data Source

This cross‐sectional study utilized national data from the Indonesian Health Survey conducted in 2023. The Indonesian Ministry of Health [25] conducted the Indonesian Health Survey 2023, a combined study of basic health research, child nutritional status, biomedical, and dental and oral health. The survey was conducted through face‐to‐face interviews and presented nationwide household data from 34,500 census blocks across 38 provinces in Indonesia. The survey primarily measured indicators such as communicable disease, noncommunicable disease, mental health and disorders, disability, pharmacy and traditional healthcare, dental and oral health, awareness and attitude, maternal health, infant and child health, nutritional status, environmental health, access to healthcare facilities, and socioeconomic status highlighting the demographic changes following the COVID‐19 outbreak [25]. Ethical considerations included ensuring anonymity and confidentiality, as well as obtaining informed consent during the interview process, all in accordance with the Law of the Republic of Indonesia Number 27 of 2022 concerning Personal Data Protection [26]. The dataset is publicly accessible on the website of the Indonesian Ministry of Health [26].

### 2.2. Study Population and Data Collection

A two‐stage stratified sampling was employed. In the first stage, census blocks were selected by sequencing urban and rural areas in each regency or city proportionally based on family size. In the second stage, a total of *n*_1_ of ordinary households and *n*_2_ of toddler households were selected through systematic sampling with implicit stratification based on the educational background of the head of the household [25]. A total of 315,646 ordinary households, 284,177 toddler households, and 1,191,692 individuals were finally included in the database.

Respondents involved in this study were individuals aged 15 years or older with hypertension and/or diabetes mellitus. Data on telemedicine usage patterns were obtained from the Telemedicine Usage Behavior section (sections G45 and G46 of the survey). Respondents with incomplete data on telemedicine use were excluded.

### 2.3. Variables and Measurements

Telemedicine utilization was assessed using a self‐report questionnaire consisting of two questions: (1) “During the last year, did you use any online healthcare services (telemedicine)?” with response options of “yes” or “no,” and (2) “If yes, please indicate what kind of services did you use?” with options including “Healthcare Service Registration, Health Education, Health Consultation, or Telepharmacy and Laboratory Examination.” Respondents who answered “no” to the first question were classified as nonusers of telemedicine.

Potential factors associated with nonuse of telemedicine services were identified based on previous studies [8, 22, 24]. These factors included age (15–65 years or older), gender (male or female), marital status (unmarried or married), education level (did not attend school, primary school, middle school, high school, or university), occupation (unemployed, government employee, self‐employed, farmer/fisherman, helper/laborer/driver, or others), type of disease (diabetes, hypertension, or both diabetes and hypertension), and island of residence (Sumatera, Java and Bali, Kalimantan, Nusa Tenggara, Sulawesi, or Maluku and Papua). The survey database provided a comprehensive list of 38 provinces in Indonesia. However, due to low statistical power, we further categorized these provinces by grouping them into these major island clusters.

### 2.4. Statistical Analysis

Given the sampling design of the Indonesian Health Survey, we applied complex sample analysis techniques prior to the analysis. To account for unequal selection probabilities, we applied the individual sampling weights provided in the dataset. This weighting procedure corrected the disproportionate representation of certain subgroups in the sample, thereby enhancing the accuracy and generalizability of the estimates to the national population. By incorporating these weights, the analysis reduces potential bias introduced by the sampling structure and yields more reliable population‐level inferences.

Descriptive statistics were used to summarize the sociodemographic information of the respondents using proportions and percentages. Bivariate logistic regression was performed to identify potential variables contributing to nonuse of telemedicine. The initial multivariate model included factors with significant associations with the nonuse of telemedicine as the outcome (*p* < 0.05). The threshold for significance for a variable to be included in the final model was set at a *p* value of 0.05. The odds ratio (OR) and 95% confidence interval (CI) were reported. The Hosmer–Lemeshow test was implemented to evaluate the fit of the final model. Furthermore, an *R*‐squared test was conducted to determine the extent to which the independent variables in the model explain the variance in the dependent variable. All statistical analyses were conducted using the Statistical Package for the Social Sciences Version 27.0.

## 3. Results

### 3.1. Respondents′ Characteristics

**Figure 1:**
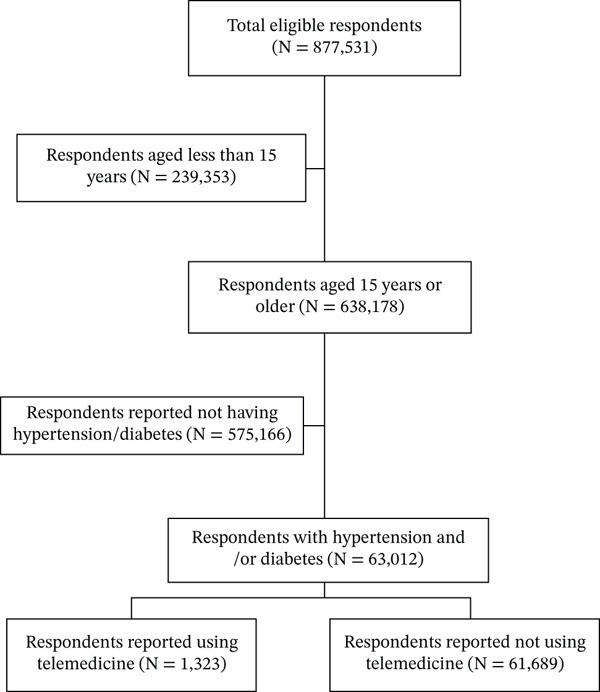
Respondent selection process.

Among the 877,531 respondents interviewed in the survey, 814,519 individuals who were under 15 years old or reported not having diabetes and hypertension were excluded. This resulted in a total of 63,012 respondents included in the study (Figure 1). As shown in Table 1, the majority of respondents were aged between 55 and 64 years old (30.9%), female (65.1%), married (78.3%), graduated from primary school (33.0%), unemployed (40.2%), lived in the islands of Java and Bali (35.8%), and had hypertension (76.3%).

**Table 1:** Respondents′ characteristics (*N* = 63,012).

Characteristics	*N* (%) Unweighted	% Weighted (95% CI)
Age in years		
15–24	262 (0.4)	0.8 (0.7–1.0)
25–34	2125 (3.4)	4.4 (4.1–4.8)
35–44	8234 (13.1)	12.5 (12.0–12.9)
45–54	17498 (27.8)	25.7 (25.1–26.3)
55–64	19488 (30.9)	29.5 (28.9–30.1)
> 65	15405 (24.4)	27.1 (26.5–27.7)
Gender		
Male	22,008 (34.9)	36.2 (35.6–36.8)
Female	41,004 (65.1)	63.8 (63.2–64.4)
Marital Status		
Not married	13,698 (21.7)	23.6 (23.0–24.2)
Married	49,314 (78.3)	76.4 (75.8–77.0)
Education		
Did not attend school	9,981 (15.8)	15.6 (15.1–16.2)
Primary school	20,798 (33.0)	35.2 (34.4–35.9)
Middle school	9,771 (15.5)	14.9 (14.4–15.3)
High school	15,439 (24.5)	24.2 (23.6–24.9)
University	7,023 (11.1)	10.1 (9.7–10.6)
Occupation		
Unemployed	25,344 (40.2)	43 (42.4–43.7)
Government employee	7,235 (11.5)	11.6 (11.2–12.1)
Self‐employed	8,469 (13.4)	14.7 (14.2–15.2)
Farmer or Fisherman	12,509 (19.9)	16.4 (15.9–17.0)
Helper/Labourer/Driver	2,910 (4.6)	6.4 (6.1–6.8)
Other	6,545 (10.4)	7.8 (7.5–8.2)
Type of disease		
Diabetes	9,344 (14.8)	14.7 (14.3–15.2)
Hypertension	48,096 (76.3)	76.3 (75.8–76.9)
Diabetes and hypertension^a^	5,572 (8.8)	8.9 (8.6–9.3)
Island of living		
Sumatera	17,453 (27.7)	17.1 (16.5–17.8)
Java and Bali	22,548 (35.8)	65 (64.1–66.0)
Kalimantan	6,779 (10.8)	6.4 (6.0–6.7)
Nusa Tenggara	3,339 (5.3)	3 (2.7–3.3)
Sulawesi	9,686 (15.4)	6.8 (6.5–7.2)
Maluku and Papua	3,207 (5.1)	1.7 (1.5–1.8)
Telemedicine utilization		
Yes	1,323 (2.0)	2.9 (2.6–3.1)
No	61,689 (98.0)	97.1 (96.8–97.4)

^a^Patient may experience both hypertension and diabetes simultaneously.

Nearly all respondents with hypertension and/or diabetes did not utilize telemedicine (98.0%). Among the 1,323 respondents who confirmed using telemedicine, the most commonly reported type of service was online healthcare service registration (75.1%), whereas 11.2% accessed clinical consultations, such as medical consultations and telepharmacy (Table 2).

**Table 2:** Type of telemedicine services used by respondents (*N* = 1,323).

No.	Type of telemedicine service	*N* (%)	% Weighted (95% CI)
1	Healthcare service registration		
	Yes	1,008 (1.6)	75.1 (70.2–79.4)
	No	315 (0.5)	24.9 (20.6–29.8)

2	Counselling and access to health information or education		
	Yes	550 (0.9)	42.6 (38.1–47.3)
	No	773 (1.2)	57.4 (52.7–61.9)

3	Clinical consultation		
	Yes	142 (0.2)	11.2 (8.8–14.3)
	No	1,181 (1.9)	88.8 (85.7–91.2)

4	Telepharmacy and laboratory or radiology examination preparation		
	Yes	109 (0.2)	8.3 (6.2–11.2)
	No	1,214 (1.9)	91.7 (88.8–93.8)

### 3.2. Factors Associated With the Nonuse of Telemedicine Among Respondents

Based on the data presented in Table 3, respondents who were older than 34 years (OR = 3.83; 95*%*CI = 1.90–7.73) and unmarried (OR = 1.40; 95*%*CI = 1.11–1.77) were more likely to not use telemedicine. Respondents who did not attend school were the least likely to use telemedicine (OR = 11.30, 95% CI = 7.20–17.75), followed by primary school graduates (OR = 9.94, 95*%*CI = 7.25–13.64), middle school graduates (OR = 4.64, 95*%*CI = 3.20–6.72), and high school graduates (OR = 2.74, 95*%*CI = 2.21–3.41), all compared with university graduates. Farmer or fisherman (OR = 2.15, 95*%*CI = 1.35–3.41) and helper, laborer, or driver (OR = 1.69, 95*%*CI = 1.05–2.72) were less likely to use telemedicine compared with those who were self‐employed. Respondents with hypertension alone had higher odds of not using telemedicine compared with those with both conditions (OR = 1.67, 95*%*CI = 1.32–2.11). Compared with respondents living in Java and Bali, those residing in Sumatera (OR = 3.11; 95*%*CI = 2.37–4.09), Kalimantan (OR = 2.34; 95*%*CI = 1.75–3.13), Nusa Tenggara (OR = 4.86; 95*%*CI = 2.96–7.99), Sulawesi (OR = 2.05; 95*%*CI = 1.46–2.86), and Maluku and Papua (OR = 4.55; 95*%*CI = 2.20–9.41) had significantly higher odds of not utilizing telemedicine.

**Table 3:** Regression logistic analysis of the nonuse telemedicine among respondents with hypertension and/or diabetes (*N* = 63,012).

No.	Characteristics	Using telemedicine % (95% CI)	Not using telemedicine % (95% CI)	Bivariate		Multivariate^c^	
				Odds ratio (95% CI)	*p*	Odds ratio^d^ (95% CI)	*p*
1	Age (in years)				< 0.001^a^		
	15–24	3.1 (1.7–5.7)	0.7 (0.6–0.9)	Ref		Ref	
	25–34	14.1 (10.5–18.7)	4.1 (3.8–4.5)	1.22 (0.58–2.54)		1.60 (0.75–3.38)	0.22
	35–44	16.8 (13.9–20.2)	12.3 (11.9–12.8)	3.04 (1.54–5.99)		3.83 (1.90–7.73)	< 0.001^b^
	45–54	26.7 (23.3–30.4)	25.7 (25.1–26.2)	3.98 (2.03–7.78)		4.30 (2.14–8.64)	< 0.001^b^
	55–64	21.9 (18.9–25.1)	29.7 (29.1–30.4)	5.64 (2.88–11.01)		5.68 (2.82–11.45)	< 0.001^b^
	> 65	17.4 (14.6–20.7)	27.4 (26.7–28.0)	6.50 (3.29–12.86)		4.68 (2.33–9.40)	< 0.001^b^

2	Gender				< 0.001^a^		
	Male	43.1 (39.1–47.3)	36 (35.4–36.6)	0.74 (0.62–0.88)		1.19 (0.95–1.48)	0.13
	Female	56.9 (52.7–60.9)	64 (63.4–64.6)	Ref		Ref	

3	Marital Status				< 0.001^a^		
	Not married	15 (12.2–18.2)	23.9 (23.3–24.4)	1.78 (1.40–2.25)		1.40 (1.11–1.77)	0.01
	Married	85 (81.8–87.8)	76.1 (75.6–76.7)	Ref		Ref	

4	Education				< 0.001^a^		
	Not attending school	3.8 (2.6–5.6)	16 (15.4–16.5)	16.37 (10.81–24.78)		11.30 (7.20–17.75)	< 0.001^b^
	Primary school	11.3 (9.0–14.2)	35.9 (35.1–36.6)	12.33 (9.38–16.21)		9.94 (7.25–13.64)	< 0.001^b^
	Middle school	12.6 (9.3–16.8)	14.9 (14.5–15.4)	4.63 (3.24–6.62)		4.64 (3.20–6.72)	< 0.001^b^
	High school	35.8 (31.9–40.0)	23.9 (23.2–24.5)	2.60 (2.14–3.15)		2.74 (2.21–3.41)	< 0.001^b^
	University	36.4 (32.5–40.5)	9.3 (8.9–9.8)	Ref		Ref	

5	Occupation				< 0.001^a^		
	Unemployed	37.5 (33.6–41.5)	43.2 (42.5–43.9)	1.48 (1.12–1.96)		1.26 (0.94–1.69)	0.12
	Government employee	30.2 (26.3–34.4)	11.1 (10.6–11.6)	0.47 (0.35–0.64)		1.00 (0.73–1.37)	0.99
	Self‐employed	18.7 (15.1–23.1)	14.5 (14.1–15.0)	Ref		Ref	
	Farmer/fisherman	3.6 (2.5–5.2)	16.8 (16.2–17.4)	6.00 (3.86–9.34)		2.15 (1.35–3.41)	< 0.001^b^
	Helper/laborer/driver	3.5 (2.4–5.1)	6.5 (6.1–6.9)	2.36 (1.49–3.74)		1.69 (1.05–2.72)	0.03
	Other	6.4 (4.9–8.3)	7.8 (7.5–8.2)	1.57 (1.09–2.28)		1.70 (1.16–2.48)	0.01

6	Type of disease				< 0.001 ^a^		
	Diabetes	17.5 (14.8–20.6)	14.6 (14.2–15.1)	1.24 (0.95–1.62)		1.31 (0.99–1.72)	0.06
	Hypertension	69.4 (65.6–72.9)	76.6 (76.0–77.1)	1.64 (1.30–2.06)		1.67 (1.32–2.11)	< 0.001^b^
	Diabetes and hypertension	13.1 (10.8–15.8)	8.8 (8.5–9.2)	Ref		Ref	

7	Island of living				< 0.001 ^a^		
	Sumatera	7.1 (5.5–9.1)	17.4 (16.8–18.1)	3.17 (2.42–4.15)		3.11 (2.37–4.09)	< 0.001^b^
	Java and Bali	83 (80.1–85.5)	64.5 (63.6–65.4)	Ref		Ref	
	Kalimantan	3.7 (2.8–4.8)	6.5 (6.1–6.8)	2.26 (1.70–3.01)		2.34 (1.75–3.13)	< 0.001^b^
	Nusa Tenggara	0.8 (0.5–1.2)	3.1 (2.8–3.3)	5.14 (3.15–8.39)		4.86 (2.96–7.99)	< 0.001^b^
	Sulawesi	4.8 (3.5–6.7)	6.9 (6.5–7.2)	1.83 (1.30–2.58)		2.05 (1.46–2.86)	< 0.001^b^
	Maluku and Papua	0.7 (0.4–1.4)	1.7 (1.5–1.8)	3.06 (1.53–6.14)		4.55 (2.20–9.41)	< 0.001^b^

Abbreviations: CI, confidence intervals and OR, odds ratios.

^a^Significant factor (*p* < 0.25)

^b^Significant factor (*p* < 0.05).

^c^Regression sig. = 0.0000; pseudo R − square test = 0.1255; Hosmer–Lemeshow test sig. = 0.3652.

^d^OR > 1 indicates higher odds of nonuse of telemedicine.

## 4. Discussion

### 4.1. Principal Results and Comparison With Prior Work

Nearly all respondents with hypertension and/or diabetes in this study did not utilize telemedicine. Factors associated with nonuse of telemedicine included being unmarried, aged older than 34 years, having an educational background below the university level, being a farmer, fisherman or helper, labourer, or driver, having hypertension, and living outside the provinces of Java and Bali.

Nearly all respondents in our study reported not using telemedicine. The low utilization of telemedicine in Indonesia may reflect disparities in digital health infrastructure across regions. Internet access, digital literacy, and device availability remain uneven between urban and rural areas. Although the Ministry of Health has initiated nationwide digital health transformation, over 80% of health facilities remain without digital systems, and many still rely on manual data recording, indicating substantial gaps in infrastructure readiness for telemedicine in Indonesia [10]. Among the small portion of respondents who used telemedicine, healthcare service registration was the most commonly used service. This trend may be attributed to the widespread adoption of telemedicine during the COVID‐19 outbreak, particularly in 2020, when many healthcare service registration procedures shifted online to ensure safer access [27, 28]. Since then, some healthcare facilities in Indonesia have implemented online registration systems.

Telemedicine utilization is generally higher in countries with higher socioeconomic levels [23]. For instance, nationwide data from the United States in 2021 indicated that 37% of adults had used telemedicine [29]. In the WHO European countries, 77% (37 out of 48) of countries offered telemedicine services, with 35% having established telemedicine programs [30]. In South Korea, over 70% of respondents had either used or planned to use telemedicine [31]. In contrast, low‐ and middle‐income countries (LMICs) showed lower adoption rates for telemedicine [23]. For example, a study in Riyadh reported that only 25.7% of 342 patients with chronic conditions had used telemedicine [32]. A study in Malaysia indicated that a lack of awareness regarding telemedicine, including its usage and benefits, may also contribute to its low utilization [33], Furthermore, environmental, individual, cultural, financial, and technological factors have all been reported as barriers to telemedicine adoption and utilization in LMICs [34].

Our findings also indicated that telemedicine was more frequently utilized by the younger age group (under 34 years). These results are corroborated by previous studies confirming this finding [31, 32, 35, 36]. Age‐related physical impairments, such as hearing or vision loss and diminished dexterity, were identified as barriers to technology adoption [37]. Additionally, significant functional impairment has been observed among older adults with hypertension or diabetes [38, 39]. Older adults often struggle with using digital devices and accessing the internet, which hampers their ability to utilize telemedicine services [40]; where more than 20 scheduled online visits were canceled due to this barrier [41]. Furthermore, less familiarity with technology and telemedicine is frequently reported among older adults [36, 42]. The reluctance to adopt telemedicine among older adults may stem from their belief that online communication cannot effectively replace in‐person care, as it lacks human touch and physical examination [36]. Addressing these barriers through education on telemedicine usage and developing user‐friendly platforms tailored for the elderly is essential to promote greater engagement. Studies have suggested that older adults who complete telemedicine visits are more likely to continue using such services, especially if they have previously established in‐person connections with their healthcare providers [36, 43].

Unmarried individuals (single, divorced, or widowed) in our study showed a higher tendency not to use telemedicine. This finding is consistent with previous research indicating that married individuals were more likely to utilize telemedicine compared with their unmarried counterparts [35]. Prior studies have shown that marital status influences healthcare utilization, as unmarried respondents displayed lower odds of having outpatient visits, including telemedicine services [44]. Furthermore, studies have reported a general survival advantage for married individuals [45], as they tend to receive more care and support from their partners in managing daily challenges related to chronic illnesses [46]. The potential lack of accountability and support from a spouse or partner may explain why unpartnered individuals are less inclined to engage in personal medical care through telemedicine.

Respondents in our study also reported nonuse of telemedicine when they had lower levels of education or occupational status. Socioeconomic disparities in telemedicine adoption are well‐documented, with numerous studies highlighting these issues [8, 31, 35, 47, 48]. Our findings indicated that individuals with lower educational attainment were less likely to utilize telemedicine services. Those with limited education may experience restricted access to technology, lower digital literacy skills, and limited flexibility in work schedules [47, 49]. Numerous studies have indicated that a higher level of education is often reflected in an individual′s career opportunity [50–52]. Higher educational attainment is associated with a transition from traditional job roles to positions that offer greater value and opportunities for advancement [53]. Aligning with these findings, our study also found that nonuse of telemedicine was more common among helpers, laborers, or drivers. The chronic conditions experienced by our study participants may further correlate with reduced employment opportunities, as indicated by previous studies [5, 54, 55]. Being a farmer or fisherman significantly increased the likelihood of not using telemedicine. This could be explained by the demographic in Indonesia, where farmers and fishermen are mostly living in rural areas. Furthermore, developing countries, including Indonesia, have struggled to ensure equal and comprehensive internet access across all regions, particularly in rural areas [56, 57]. Moreover, ownership of digital devices and the ability to use them may also impact telemedicine utilization in these regions [58]. As a result, access to telemedicine may be limited for this population, leading to decreased engagement with such services.

Our findings indicated that patients managing a single chronic condition, such as hypertension, were less likely to use telemedicine services compared with those managing both conditions simultaneously. This suggests that individuals with more complex health needs requiring ongoing monitoring are more likely to seek digital healthcare services. This pattern is consistent with previous research indicating that individuals with chronic illnesses are more likely to adopt telemedicine due to the necessity for regular consultations and continuous disease management [59, 60]. This relationship may be attributed to the increased reliance on telemedicine during the COVID‐19 pandemic, particularly for patients at higher risk of severe outcomes. During the pandemic, individuals with chronic diseases were encouraged to minimize in‐person healthcare visits and instead use telemedicine services for monitoring their conditions telemedicine [61]. This increased exposure likely fostered familiarity and positive attitudes toward telemedicine, making it a preferred option for managing long‐term health needs [62]. Conversely, individuals without chronic conditions or those managing a single condition may not perceive an immediate need for telemedicine services, particularly if their condition requires minimal ongoing monitoring. Their lower engagement could be due to fewer healthcare interactions or a lack of urgency in seeking medical advice, thereby limiting their exposure to and familiarity with telemedicine platforms [63]. Addressing this gap in telemedicine utilization requires tailored interventions to increase awareness and demonstrate the benefits of digital healthcare, even for those with less complex health needs.

Our study also suggested that respondents living outside the islands of Java and Bali were more likely not to use telemedicine compared with those living in these provinces. One possible explanation is that both the completion rate for education and internet penetration in Java and Bali are significantly higher than that in other provinces [64]. Indonesia, with its archipelagic geography comprising over 17,000 islands and a population exceeding 270 million, faces challenges in addressing the digital divide. Provinces on Java Island exhibit superior digital connectivity, academic institutions, and advanced infrastructure [65]. Notably, nearly 90% of the population in Java and Bali have access to the internet [66], facilitating easier access to telemedicine services. Meanwhile, regions such as Kalimantan, Sumatera, Papua, and Maluku struggle with internet access and generally have lower digital capabilities, despite recent improvements [67]. Therefore, strategies aimed at bridging the digital divide, such as investing in broader internet coverage, providing training in technology use, and educating the public to improve attitudes towards technology [68], are important in ensuring equal access to telemedicine for chronic patients. The high proportion of respondents using telemedicine primarily for online service registration reflects a pattern in which telemedicine functions more as a digital administrative tool rather than a platform for clinical interaction. This finding may highlight early‐stage adoption of telemedicine in Indonesia, where logistical convenience is utilized first before clinical services are adopted.

### 4.2. Clinical and Policy Implications

The findings of this study contribute to the current understanding of factors affecting the nonuse of telemedicine. Interventions aimed at improving chronic patients′ familiarity with telemedicine, especially among older patients, could enhance their engagement with these services [36]. This can be facilitated by developing user‐friendly telemedicine services tailored for older adults [69] and by providing training to facilitate technology adoption [70], either through community health centers and primary care clinics.

To address digital inequality, especially in regions with inadequate internet access, establishing freely accessible telemedicine points, for example, in community health centers could serve as an initial step [71]. In addition, policymakers could consider subsidizing internet/data packages for low‐income or rural patients to reduce financial barriers to access. Integrating telemedicine training into medical, pharmacy, and nursing curricula, as well as incorporating digital health topics into school education [72], may help bridge long‐term digital capability gaps across generations. From a service‐delivery perspective, telemedicine should be incorporated into routine primary care workflows, for example by linking teleconsultations with electronic medical records, prescription systems, and follow‐up appointment scheduling. Clinical staff should be supported through training and clear guidelines to ensure telemedicine becomes part of standard care rather than an optional service [73].

To effectively expand telemedicine on a national scale, collaborative efforts among individuals, communities, healthcare providers, and policymakers are essential [73]. Policymakers and healthcare providers should implement supportive regulations, provide financial incentives where appropriate, and ensure that telemedicine platforms comply with data privacy, security, and clinical safety standards [74].

### 4.3. Strengths and Limitations

To the best of our knowledge, this study is the first to examine the factors associated with the nonuse of telemedicine among patients with diabetes or hypertension in Indonesia. A significant strength of this study is the use of a nationwide database from the 2023 Indonesian Health Survey that covered 38 provinces in Indonesia and had an average response rate of 98.74%. This survey allowed for favorable conditions, including representation across Indonesia, a high sample size, and relative heterogeneity. Additionally, we applied complex sample analysis techniques using survey weights for accurate estimation and correction of the unequal sampling proportions. However, the study has some limitations. Since this research was a cross‐sectional study, it does not allow for causal assumptions about the relationship between the nonuse of telemedicine and associated factors. Secondly, telemedicine utilization was assessed using a single self‐reported yes/no item that does not differentiate between individuals who used telemedicine only once and those who used it regularly. As such, the measure may not fully capture the frequency, intensity, or continuity of telemedicine use, which could introduce misclassification bias and limit interpretation of the findings. Future studies should employ more detailed measures of telemedicine use such as frequency and duration of telemedicine service to better capture utilization patterns. Thirdly, the prevalence of telemedicine use in this study was low (2%, *n* = 1,323), which may limit the statistical power of the regression analyses and contribute to wide confidence intervals. Therefore, the associations observed should be interpreted with caution. Furthermore, the reliance on self‐reported data may introduce recall bias, whereby respondents may not accurately remember or report past events. Lastly, some important predictors of telemedicine use, such as household income, smartphone ownership, digital literacy, health insurance coverage, and internet availability were not captured in the survey. The absence of these well‐established determinants of digital health may limit the completeness of our model and partially explain residual confounding. Future studies should include these variables to provide a more comprehensive analysis of telemedicine utilization.

### 4.4. Future Research

Future studies should incorporate qualitative or mixed methods approaches to explore users and provider perspectives on telemedicine services, including perceived barriers, facilitators, usability challenges, and cultural factors influencing adoption. These insights can help improve the relevance and effectiveness of telemedicine programs. In addition, more sophisticated analytical approaches, such as principal components, should be considered. Furthermore, it would be beneficial for future research to include data on trends in telemedicine usage among different socioeconomic groups, focusing not only on utilization patterns but also on patient experiences and the usability of existing telemedicine services.

## 5. Conclusions

The utilization rate of telemedicine among patients with diabetes and/or hypertension in Indonesia is relatively low. Several sociodemographic characteristics, including being unmarried, older age, having a low educational background, having low occupational status, having hypertension, and living outside the islands of Java and Bali, are associated with a higher likelihood of not using telemedicine. Personalized approaches by considering patient‐specific factors, along with more frequent integration of telemedicine into the healthcare system, are essential for increasing telemedicine adoption and improving chronic disease management in Indonesia.

**Nomenclature**
CI: Confidence interval
OR: Odds ratio

## Author Contributions

S.D.A. and M.A.A.P. conceived and designed the analysis. S.D.A., M.A.A.P., R.M.F., W.N.I., and R.A. are involved in data analysis. S.D.A., M.A.A.P., and M.K. are involved in data interpretation. S.D.A. and M.G. drafted the manuscript. All authors made critical revisions of the manuscript.

## Funding

No funding was received for this manuscript.

## Disclosure

All authors approved the final version of the manuscript.

## Conflicts of Interest

The authors declare no conflicts of interest.

## Supporting information


**Supporting Information** Additional supporting information can be found online in the Supporting Information section. Table S1 STROBE assessment (Word file).

## Data Availability

The data used in this study are publicly available from the 2023 Indonesia Health Survey (SKI). The data can be accessed at https://www.badankebijakan.kemkes.go.id/hasil-ski-2023/.
